# Endocrine disruptors, obesity, and metabolic syndrome

**DOI:** 10.55730/1300-0144.6120

**Published:** 2025-10-26

**Authors:** Tuğba BARLAS, Alev EROĞLU ALTINOVA, Meriç COŞKUN, Ethem Turgay CERİT

**Affiliations:** Division of Endocrinology and Metabolism, Department of Internal Medicine, Faculty of Medicine, Gazi University, Ankara, Turkiye

**Keywords:** Bisphenols, phthalates, tributyltin, insulin resistance, metabolic syndrome, obesity

## Abstract

The global prevalence of obesity and metabolic syndrome (MetS) is rising worldwide, and increasing evidence suggests that chemical exposures—particularly endocrine disruptors (EDs)—represent a significant contributing factor. EDs can act as obesogens, increasing the risk of weight gain and related metabolic conditions, including type 2 diabetes, dyslipidemia, hypertension, and cardiovascular disease. They may also alter the basal metabolic rate, gut microbiota composition, and hormonal regulation of appetite and satiety. EDs are reported to exert their effects mainly through the peroxisome proliferator-activated receptor gamma pathway, which is primarily expressed in adipose tissue and is a key regulator of adipogenesis. Common consumer products such as plastic bottles, metal food cans, detergents, toys, cosmetics, and pesticides frequently contain EDs. Humans can be exposed to these chemicals via multiple routes, including transplacental transfer, breast milk, inhalation, ingestion, and dermal absorption. Bisphenols, tributyltin, phthalates, per- and polyfluoroalkyl substances, polycyclic aromatic hydrocarbons, and heavy metals are among the known EDs that have been associated with obesity and MetS. The need for further investigation and stricter regulations to mitigate the public health consequences of environmental exposure to EDs is consistently emphasized in recent literature. Understanding the mechanisms by which EDs affect various hormones and systems is essential for developing effective prevention and intervention strategies. In this review, we discuss the relationship between obesity, MetS, and EDs, along with exposure pathways and preventive strategies.

## 1. Introduction

The global prevalence of obesity is rising rapidly. Over 600 million individuals, including approximately 40 million children under the age of 5, currently suffer from obesity. Furthermore, studies indicate that approximately 80% of children with obesity remain affected into adulthood [1]. Numerous studies have demonstrated that obesity adversely impacts both life expectancy and quality of life [2]. Although lifestyle factors such as diet and physical activity are major contributors, growing evidence suggests that other factors, particularly chemical exposures, may also play a role [3]. Chemicals known as “obesogens” are thought to promote weight gain by affecting the endocrine system, which regulates metabolism, energy balance, and appetite. This leads to excess body fat and associated adverse health outcomes [4,5]. Animal studies have demonstrated that exposure to endocrine disruptors (EDs) during early life increases susceptibility to weight gain and metabolic comorbidities, including alterations in lipid metabolism, type 2 diabetes (T2D), and cardiovascular disease [6]. Additionally, recent literature has identified associations between arterial hypertension and specific EDs [7]. Obesity and related comorbidities together constitute metabolic syndrome (MetS), which is considered a strong predictor of cardiovascular morbidity and mortality [8]. MetS is defined as the presence of at least three of the following five clinical criteria: (I) increased waist circumference; (II) elevated triglyceride levels; (III) decreased HDL concentrations; (IV) high blood pressure; (V) elevated fasting glucose levels [9]. The reported prevalence of MetS varies according to the diagnostic criteria, ranging from 12.5% to 31.4% [10].

This review aims to provide an overview of EDs in relation to obesity and MetS, focusing on molecular mechanisms and newly recognized aspects such as mixture interactions, gut microbiota alterations, and multigenerational effects. Building on previous reviews, this paper emphasizes new mechanistic evidence and preventive approaches, linking exposure pathways, chemical groups, and regulatory measures to broader clinical and public health implications.

## 2. Materials and methods

A narrative literature search was conducted using the PubMed, Scopus, and Web of Science databases, focusing on studies published within the past 15 years—a period marked by a notable rise in ED-related research. The search terms included “endocrine disruptors”, “obesogens”, “obesity”, “metabolic syndrome”, and specific chemical groups such as “bisphenols”, “phthalates”, “PFAS”, “pesticides”, and “heavy metals”. The titles and abstracts were independently screened by two authors, and full-text articles addressing the relationship between EDs, obesity, and MetS were included. Human and experimental studies, review articles, and regulatory reports were all included to ensure a comprehensive overview. Studies were excluded if they were not available in English, if the full text could not be accessed, or if they did not directly address the relationship between EDs and obesity or MetS. Conference abstracts, editorial letters, and commentaries were also excluded.

## 3. Endocrine disruptors

Endocrine disruptors are exogenous substances that adversely affect the endocrine system by interfering with hormonal activity at specific doses [11]. According to the U.S. Environmental Protection Agency, EDs are agents that disrupt the synthesis, secretion, transport, binding, or elimination of hormones in the body [12]. These hormones play essential roles in maintaining homeostasis, regulating reproduction and development, and influencing behavior. Consequently, an endocrine-disrupting substance is any compound—natural or synthetic—that can interfere with the normal hormonal functions through environmental exposure [13].

According to the Endocrine Society’s scientific statement, EDs are highly diverse, encompassing synthetic chemicals used as industrial solvents and lubricants, along with their byproducts such as polychlorinated biphenyls (PCBs), per- and polyfluoroalkyl substances (PFAS), polybrominated biphenyls (PBBs), and dioxins. They also include plastics such as bisphenol A (BPA), plasticizers like phthalates, pesticides including methoxychlor, chlorpyrifos, and dichlorodiphenyltrichloroethane, fungicides such as vinclozolin, and pharmaceutical agents like diethylstilbestrol. Additionally, naturally occurring compounds found in human and animal diets—such as phytoestrogens including genistein and coumestrol—can also act as EDs [14].

## 4. Exposure to endocrine disruptors

EDs may be present in a wide range of commonly used products, including plastic bottles, metal food cans, detergents, flame retardants, toys, cosmetics, and pesticides [15]. Humans are often exposed to multiple environmental chemicals simultaneously rather than to a single compound. The term “cocktail effect of Eds” refers to the combined consequences of such exposure. Combining chemicals can result in additive, antagonistic, or synergistic effects. These interactions may be stronger or qualitatively different from the effects of each individual chemical [16].

Humans may be exposed to these substances via transplacental transfer, breast milk, inhalation, ingestion, or dermal absorption [17]. Exposure to EDs begins during the neonatal period and persists throughout life. Exposure to EDs is particularly prevalent during critical stages such as gestation, infancy, and early childhood, increasing the likelihood of disease development later in life and potentially impacting future generations [18]. This increased risk arises because fetuses and infants experience greater tissue exposure than adults. In addition, they have lower levels of cytochrome P450 enzymes that metabolize xenobiotics [19]. Exposure to obesogenic substances during pregnancy or early-life lactation can disrupt critical physiological processes, including energy metabolism, appetite regulation, and adiposity development [20].

Multigenerational effects of EDs occur when exposure in utero leads to transmission to subsequent generations, persisting across several generations [21,22]. For an effect to be classified as transgenerational, it must manifest in individuals who were never directly exposed to EDs [23,24]. Transgenerational effects of EDs are usually mediated through epigenetic changes. DNA methylation, histone modifications, and the involvement of noncoding RNAs constitute key mechanisms underlying epigenetic alterations in the germline [25,26]. For instance, prenatal exposure to tributyltin in mice has been shown to increase adipose tissue mass and adipocyte size, as well as induce fatty liver across three subsequent generations [27]. The mechanisms underlying the transmission of ED effects across generations remain poorly understood, warranting further investigation—particularly in human studies. A deeper understanding of these multigenerational and transgenerational effects may help mitigate their adverse impacts on human health.

## 5. Most common endocrine disruptors in relation to obesity and metabolic syndrome

### 5.1. Bisphenols

Bisphenol A (BPA) was synthesized through the condensation of acetone with phenol [28]. BPA is currently used extensively in the production of epoxy resins and polycarbonate plastics, which are found in various food and beverage storage products such as bottles, containers, and cans [29]. Dietary intake accounts for approximately 99% of total BPA exposure, as these plastics can leach small amounts of BPA into stored foods and beverages [29]. BPA has a biological half-life of approximately 6 h in humans, with peak plasma concentrations occurring within 80 min following oral administration. [30]. Studies have identified BPA and its metabolites in the urine of approximately 92.6% of the population [31]. BPA has also been detected in breast milk and amniotic fluid, indicating that exposure begins in utero and persists postnatally [32]. Currently, there is no globally standardized acceptable limit for BPA exposure [33]. The former temporary tolerable daily intake (TDI) of 4 μg/kg body weight/day has been revised to 0.2 ng/kg body weight/day for BPA exposure by the European Food Safety Authority (EFSA) [34].

Recent metaanalyses have indicated a significant relationship between exposure to BPA and obesity in both children and adults [35,36]. Some studies have suggested a stronger association between bisphenol analogues such as bisphenol F (BPF) and obesity, particularly among boys [37]. Another study reported no association between BPA and obesity; however, bisphenol analogues such as bisphenol S (BPS) and BPF were linked to obesity [38]. BPA has also been linked to MetS in the literature [39]. In a cross-sectional study of 2104 participants from the NHANES database, a positive correlation was observed between BPA exposure and the risk of MetS, independent of potential confounders such as age, sex, ethnicity, smoking, alcohol consumption, physical activity, and urinary creatinine levels [40].

BPA and its analogues (e.g., BPF, BPS) are thought to act as obesogens through multiple molecular mechanisms that disrupt metabolic homeostasis. A major pathway involves direct activation of peroxisome proliferator-activated receptor gamma (PPAR-γ), the key transcription factor regulating adipocyte differentiation. PPAR-γ promotes adipocyte differentiation and induces the expression of enzymes involved in lipid synthesis [41]. It also maintains metabolic homeostasis by regulating genes associated with energy balance. Recent studies confirmed that BPA can bind to the PPAR-γ ligand-binding domain, enhancing adipogenesis and lipid accumulation [42,43]. Furthermore, due to its hormone-like properties, BPA can bind to estrogen receptors. Although traditionally regarded as a weak estrogen compared with 17β-estradiol, BPA can exert potent endocrine-disrupting effects even at very low concentrations, acting through both classical genomic and rapid nongenomic signaling pathways. These actions influence not only adipose tissue function but also body weight regulation, cardiovascular physiology, and reproductive health [44]. BPA has also been shown to impair pancreatic β-cell function and insulin secretion, contributing to glucose intolerance and insulin resistance [45]. Epigenetic modifications represent another key mechanism: BPA exposure alters DNA methylation of metabolic genes, including the PPAR-γ promoter, potentially resulting in persistent adipogenic programming and transgenerational effects [46]. In addition, experimental studies have demonstrated that BPA increases oxidative stress and induces chronic low-grade inflammation in adipose tissue, both of which are central drivers of insulin resistance and cardiometabolic dysfunction [47]. Through these converging molecular pathways, bisphenols may substantially contribute to obesity, dyslipidemia, hypertension, and other key features of metabolic syndrome.

Although the association between bisphenols and glucose metabolism impairment is well established, data on their effects on lipid metabolism remain inconclusive [48,49]. Furthermore, several studies have linked BPA exposure to the development of hypertension [50,51]. In conclusion, findings from observational studies suggest that BPA exposure may be a risk factor for obesity and several components of MetS.

### 5.2. Tributyltin

Tributyltin (TBT) is one of the most extensively studied obesogens [52]. It is primarily used in the marine industry to prevent the growth of algae and other marine organisms. It can also be found in pesticides [52,53]. The primary sources of human exposure to TBT include the consumption of contaminated seafood, occupational contact in industries utilizing TBT-containing products, and exposure to contaminated water. [53].

TBT exposure promotes adipocyte differentiation by activating PPAR-γ and retinoid X receptor [33]. In vivo studies have demonstrated that TBT exposure induces the differentiation of preadipocytes, resulting in dysfunctional adipocytes with altered lipid metabolism and gene expression [54]. Additionally, TBT exposure has been associated with fat accumulation and the development of hepatic steatosis in snails, fish, and rodents [55,56]. Numerous studies have shown that prenatal exposure to TBT leads to increased adipose tissue deposition in offspring, with these effects persisting across generations. This suggests that epigenetic mechanisms may play a role in these outcomes [57,58].

### 5.3. Phthalates

Phthalates are one of the most commonly used plasticizers worldwide, with a yearly consumption of 7.5 million tons [39]. Food packaging, vinyl flooring, detergents, lubricants, adhesives, automotive plastics, children’s toys, textiles, and wallpapers are among the many products that contain phthalates [59].

The literature reports associations between phthalate exposure and obesity-related factors, glucose dysregulation, and hypertension [60–62]. In children, a systematic review and metaanalysis revealed significant associations between individual phthalate metabolites and body mass index (BMI), waist circumference, and serum glucose levels [60]. Some studies have also reported associations between phthalate exposure and childhood hypertension, although one study found no significant relationship between phthalate metabolites and lipid parameters such as triglycerides or high-density lipoprotein levels in children and adolescents [63,64].

Phthalate exposure is increasingly associated with metabolic disorders; however, the precise mechanisms underlying these effects remain unclear. Phthalates may promote the differentiation of preadipocytes into mature adipocytes and enhance intracellular fat storage by activating PPAR-γ, a key regulator of adipogenesis and adipose tissue function [65]. In liver tissue, phthalates also stimulate the constitutive androstane receptor, which plays an important role in xenobiotic metabolism and can modulate lipid and glucose homeostasis, contributing to hepatic steatosis and systemic insulin resistance [66]. Furthermore, phthalate exposure induces oxidative stress, mitochondrial dysfunction, and endoplasmic reticulum stress in hepatocytes, thereby impairing metabolic signaling [67]. These alterations may enhance gluconeogenesis, inhibit fatty acid oxidation, and exacerbate hepatic lipid accumulation, ultimately contributing to features of metabolic syndrome.

### 5.4. Per- and polyfluoroalkyl substances (PFAS)

Per- and polyfluoroalkyl substances (PFAS) are a group of synthetic chemicals valued for their resistance to water, oil, and heat. They are found in a wide range of products, including nonstick cookware, water-repellent fabrics, food packaging, and firefighting foams. PFAS are also used in industrial processes such as electronics manufacturing and metal plating [68]. Due to their environmental persistence, PFAS can bioaccumulate in ecosystems and the human body, leading to potential health risks including endocrine disruption [69].

A recent review reported associations between PFAS exposure and the development of obesity, diabetes, and metabolic dysfunction-associated steatotic liver disease (MASLD) [70]. Furthermore, a study investigating MetS outcomes among mother–child pairs exposed to perfluorooctanoate (PFOA) through drinking water found that perfluorononanoic acid was associated with increased MetS risk, greater waist circumference, elevated triglyceride levels, and reduced high-density lipoprotein (HDL) concentrations [71]. Another notable finding from that study was that concentrations of both PFOA and perfluorooctane sulfonate (PFOS) were higher in children than in their mothers, persisting until approximately age 12 for PFOA and age 19 for PFOS [71].

The mechanisms linking PFAS to MetS are complex and not yet fully understood. Most studies have focused on PFOA and PFOS. Evidence from animal and human studies indicates that PFAS activate peroxisome proliferator-activated receptor alpha (PPAR-α) and, to a lesser extent, PPAR-γ, thereby disrupting lipid and glucose metabolism and promoting adipogenesis [72,73]. They can also interfere with thyroid hormone transport and signaling, as PFOS and PFOA competitively bind to transthyretin, potentially disrupting systemic thyroid regulation [74]. In addition, PFAS exposure disrupts bile acid metabolism and promotes hepatic lipid accumulation, thereby linking these compounds to MASLD [75].

### 5.5. Polychlorinated biphenyls (PCBs) and polybrominated biphenyls (PBBs)

PCBs have been extensively used in industry due to their electrical insulation properties, high boiling point, chemical stability, and resistance to fire. PBBs were primarily used as flame retardants in various consumer products to minimize fire risk [76]. Several studies have highlighted that exposure to PCBs and PBBs may be associated with an increased incidence of cardiovascular disease, endothelial and endocrine dysfunction, hypertension, and hyperlipidemia [77–79].

Similar to PFAS, PCBs and PBBs are persistent organic pollutants that accumulate in adipose tissue, disrupt thyroid hormone signaling, and induce oxidative stress and inflammation, thereby contributing to insulin resistance and metabolic dysfunction [72,75]. In addition, they may activate the aryl hydrocarbon receptor, thereby altering xenobiotic metabolism and further disturbing lipid and glucose homeostasis [80].

### 5.6. Polycyclic aromatic hydrocarbons (PAHs)

Polycyclic aromatic hydrocarbons (PAHs) are carbon- and hydrogen-based pollutants that are ubiquitous in the atmosphere, water, and soil. They are formed through the incomplete combustion of coal, oil, gas, vehicle emissions, and tobacco smoke. Common household products such as cosmetics, coatings, and rubber materials may also contain PAHs. Although inhalation is the primary route of human exposure, ingestion and dermal absorption can also occur [81]. Increased waist circumference and obesity in children have been positively associated with total urinary PAH and naphthalene metabolites [82]. Benzo[a]pyrene, a representative PAH, has been reported to inhibit lipolysis and increase fat deposition in adult mice [83]. PAHs undergo metabolic activation via cytochrome P450 enzymes to form reactive metabolites that generate oxidative stress and DNA adducts. This oxidative and inflammatory burden contributes to adipocyte dysfunction, insulin resistance, and endothelial injury [84]. Consequently, several studies have demonstrated associations between PAH exposure and T2D, hypertension, and dyslipidemia [85–87].

### 5.7. Pesticides

Current literature indicates that global pesticide production reached 3.5 million tons in 2020 [88]. Organophosphates, carbamates, organochlorine pesticides, pyrethroids, and triazines are among the most extensively studied classes of pesticides [89]. Each of these pesticide classes exerts distinct adverse effects on human health. However, in real-life scenarios, humans are seldom exposed to a single chemical agent. Instead, humans are simultaneously exposed to multiple pesticides and various other environmental contaminants. This phenomenon, often referred to as the “cocktail effect”, describes the synergistic or additive interactions among multiple chemicals. Even if the concentration of each pesticide is below its individual toxic threshold, their combined presence can disrupt endocrine and metabolic pathways, amplify oxidative stress, and worsen inflammatory responses. Such combined exposures have been shown to impair insulin signaling, alter lipid metabolism, and contribute to adipocyte dysfunction. Consequently, simultaneous exposure to multiple pesticides and contaminants may increase the risk of T2D, dyslipidemia, and insulin resistance through the so-called “cocktail effect” [90]. A recent metaanalysis reported that overall pesticide exposure was associated with a 42% increase in the risk of metabolic syndrome. It has also been reported that pesticides accumulate in adipose tissue, and their adverse effects may intensify with increasing BMI [89]. Current evidence in the literature primarily focuses on organochlorine pesticide exposure, indicating the need for further research on other pesticide classes [89].

### 5.8. Heavy metals

Heavy metals can enter the human body both directly and indirectly, accumulating over time through the consumption of food and water or via inhalation [91]. Heavy metals can disrupt normal endocrine function, induce oxidative stress, and initiate inflammatory responses. These effects play an essential role in the development of obesity and MetS [92]. A recent review suggested that the four most concerning heavy metal pollutants—arsenic, cadmium, lead, and mercury—may share common mechanistic pathways contributing to MetS development. These metals can promote mitochondrial dysfunction, disrupt adipokine secretion, and impair insulin signaling, thereby contributing to metabolic dysregulation [93]. Furthermore, studies have shown that the prevalence of MetS is higher among individuals exposed to heavy metals [94]. Studies have revealed that leptin levels in the serum of offspring from pregnant rodents and women—as well as in placental tissue and cord blood—significantly increase following exposure to arsenic-contaminated water, as demonstrated in both animal and human studies [95–97]. In addition, in a systematic review, Tinkov et al. analyzed six studies on obesity, five of which reported an association between mercury exposure and increased obesity risk [98].

## 6. Additional pathophysiological mechanisms of endocrine disruptor–induced metabolic dysfunction

In addition to the specific mechanisms described for each chemical group, several shared biological pathways are believed to contribute to the metabolic effects of EDs. One of these involves interference with neuroendocrine signaling, which can influence appetite regulation, food preference, and satiety control [33]. The hypothalamus, a key brain region regulating feeding behavior, can also be disrupted by ED exposure [14]. Differences in eating behavior between normal-weight and obese individuals may result from hypothalamic dysfunction, which could alter metabolic set points, particularly during adolescence and adulthood [99,100]. The structure and function of dopamine pathways in the developing brain can also be altered by exposure to EDs. For instance, early exposure to BPA has been shown to alter dopaminergic activity in brain regions associated with impulsive and addictive behaviors [101]. Another human study reported that BPA levels were inversely associated with ghrelin and positively correlated with leptin and adiponectin, key hormones regulating glucose, lipid metabolism, and satiety [102].

Furthermore, EDs can disrupt energy homeostasis by facilitating caloric accumulation through alterations in basal metabolic rate, gut microbiota composition, and nutrient storage [4,103,104]. These changes may reduce overall energy expenditure and promote lipid deposition in adipose tissue, contributing to insulin resistance. EDs influence energy expenditure through their effects on brown adipose tissue activity, skeletal muscle metabolism, and the synthesis and action of thyroid hormones. Suppression of thermogenic activity in brown adipose tissue and decreased mitochondrial respiration can impair heat generation and energy utilization [105].

Exposure to EDs also induces several alterations, including the activation of molecular pathways and disruption of intestinal microbial homeostasis. Dysbiosis may alter the balance of beneficial and pathogenic bacteria, leading to increased intestinal permeability, endotoxin release, and chronic low-grade inflammation. The host can absorb metabolites produced through microbial degradation of EDs. Some of these microbial metabolites—such as short-chain fatty acids, secondary bile acids, and lipopolysaccharides—can disrupt lipid and glucose metabolism, thereby exacerbating oxidative stress and inflammatory signaling [106].

Current evidence on the metabolic effects of endocrine disruptors remains heterogeneous and, in some cases, contradictory. Most existing studies are observational and have relatively short follow-up durations, which limits causal inference. Furthermore, humans are seldom exposed to a single compound, and multiple concurrent exposures make it challenging to determine the individual contribution of each chemical. These limitations should be considered when interpreting findings and underscore the need for long-term, well-designed studies to elucidate both the independent and combined effects of EDs on metabolic health.

## 7. Prevention strategies to reduce the adverse effects of endocrine disruptors

Increasing awareness of the sources and hazards of EDs and promoting public engagement are essential to mitigate their adverse health effects. Although individual actions are limited, minimizing exposure by avoiding products known to contain EDs remains crucial. The most critical measure, however, is for authorities to implement effective preventive strategies and enforce appropriate regulations.

Recent regulatory examples highlight the importance of stronger preventive measures. As mentioned previously, EFSA recently reduced the TDI for BPA by nearly 20,000-fold, underscoring the growing recognition of its potential risks [34]. However, discrepancies persist among regulatory bodies; for instance, the European Medicines Agency has raised formal objections to several aspects of EFSA’s proposal [107]. This divergence highlights the need for closer collaboration among policymakers and international agencies to develop coherent and effective preventive strategies.

Metaanalyses have also demonstrated that pesticide exposure increases the risk of MetS by approximately 42% [89], supporting the need for stricter monitoring and regulation. In response to strong evidence linking PFAS with obesity, diabetes, and dyslipidemia, several countries have established drinking water standards and monitoring programs^1^ [108].

Practical measures may include substituting BPA with safer alternatives, restricting the use of phthalates in food packaging, reducing pesticide residues in agricultural products, and ensuring regular monitoring of PFAS contamination in water sources. Additional strategies involve advancing detection technologies, applying standardized methodologies for exposure assessment, encouraging cross-sectoral collaboration, and incorporating scientific research into policy development [109]. Consequently, reducing exposure through increased public awareness, education, evidence-based environmental policies, and strengthened occupational safety measures is essential to mitigate the health risks associated with EDs.

## 8. Conclusion

Current evidence suggests that EDs contribute to the development of obesity and MetS by disrupting hormonal balance, altering adipogenesis, and impairing insulin signaling. Additionally, the “cocktail effect” of combined exposures, transgenerational epigenetic modifications, and gut microbiota dysregulation provide insight into how even low-dose and lifelong exposures can lead to persistent health consequences. These findings highlight that EDs not only affect adiposity but also exacerbate key components of MetS, such as dyslipidemia, hypertension, and glucose intolerance. From a public health standpoint, stricter regulations, continuous monitoring, and targeted preventive strategies—particularly for vulnerable populations—are essential to minimize exposure and reduce the global burden of ED-associated metabolic disorders.

## Footnotes

1 United States Environmental Protection Agency. Biden-Harris Administration Finalizes First-Ever National Drinking Water Standard to Protect 100M People from PFAS Pollution. 2024. Web site: https://www.epa.gov/newsreleases/biden-harris-administration-finalizes-first-ever-national-drinking-water-standard
